# Unbiased subgenome evolution following a recent whole-genome duplication in pear (*Pyrus bretschneideri* Rehd.)

**DOI:** 10.1038/s41438-018-0110-6

**Published:** 2019-03-01

**Authors:** Qionghou Li, Xin Qiao, Hao Yin, Yuhang Zhou, Huizhen Dong, Kaijie Qi, Leiting Li, Shaoling Zhang

**Affiliations:** State Key Laboratory of Crop Genetics and Germplasm Enhancement, Centre of Pear Engineering Technology Research, Nanjing Agricultural University, 210095 Nanjing, China

**Keywords:** Plant evolution, Plant genetics

## Abstract

Genome fractionation (also known as diploidization) frequently occurs following paleopolyploidization events. Biased fractionation between subgenomes has been found in some paleo-allopolyploids, while this phenomenon is absent in paleo-autopolyploids. Pear (*Pyrus bretschneideri* Rehd.) experienced a recent whole-genome duplication (WGD, ~30 million years ago); however, the evolutionary fate of the two subgenomes derived from this WGD event is not clear. In this study, we identified the two paleo-subgenomes in pear using peach (*Prunus persica*) as an outgroup and investigated differences in the gene loss rate, evolutionary rate, gene expression level, and DNA methylation level between these two subgenomes. Fractionation bias was not found between the two pear subgenomes, which evolved at similar evolutionary rates. The DNA methylation level of the two subgenomes showed little bias, and we found no expression dominance between the subgenomes. However, we found that singleton genes and homeologous genes within each subgenome showed divergent evolutionary patterns of selective constraints, expression and epigenetic modification. These results provide insights into subgenome evolution following paleopolyploidization in pear.

## Following a trail of duplicated pear DNA

Genomic sleuthing reveals insights into an ancestral chromosomal duplication event from the early evolution of the pear tree. Such ‘whole-genome duplication’ is common among plants, and can arise from the combination of genetic material of plants from either the same (autopolyploid) or different (allopolyploid) species. The pear is known to have undergone a duplication event 30 million years ago, and Shaoling Zhang and colleagues at Nanjing Agricultural University in China set out to assess the nature of this genome expansion. After analysis of pear genome data and comparison against other fruit plant species, the researchers determined that the pear is an autopolyploid, and that genes found in just one ‘subgenome’ have subsequently evolved expression profiles that differ noticeably from those genes that are present as multiple homologues amongst the duplicated chromosomes.

## Introduction

The evolution of plant genomes has been influenced by frequent occurrences of ancient whole-genome duplication (WGD; also known as paleopolyploidization), which provide abundant genetic material for survival, phenotypic diversification and radiation of plants^1–6^. Polyploidization commonly occurs through autopolyploidization or allopolyploidization, generating two forms of polyploid: autopolyploids and allopolyploids^7^. Extensive genome fractionation (also known as diploidization) occurs following WGD, reverting the polyploids to stable diploid status^6,8,9^. Subgenome dominance, characterized by bias in gene loss, gene expression and DNA methylation between two subgenomes derived from polyploidization, has been observed in paleo-allopolyploids such as *Arabidopsis thaliana*, maize (*Zea mays*), Chinese cabbage (*Brassica rapa*) and *Brassica oleracea*^10–12^. The dominant subgenome often retains more ancestral genes, which show expression dominance, while the submissive subgenome has fewer ancestral genes, with reduced expression of surviving genes^13^. However, subgenome dominance is absent in paleo-autopolyploids such as poplar (*Populus trichocarpa*)^14^ and even some paleo-allopolyploids, such as soybean (*Glycine max*)^15^ and cucurbits (*Cucurbita maxima* and *Cucurbita moschata*)^16^.

The phenomenon of biased fractionation between two paleo-subgenomes was first uncovered in maize^12^. However, investigation of diverse maize inbred lines found that differential fractionation among individuals is rare^17^. Many previous studies have sought to dissect the mechanisms driving biased fractionation between subgenomes. An association between biased gene lossv and biased gene expression between subgenomes has been revealed^18,19^. The genes retained in the overfractionated subgenome tend to have lower expression levels and contribute less to phenotypic variation, eventually resulting in their loss owing to weak impact on fitness^12,19^. Reduction in gene expression, which may be caused by relaxed selective pressure imposed on genes and elevated methylation level, leads to a high rate of fractionation in the overfractionated subgenome^19,20^. In *B. oleracea* and *B. rapa*, three distinct subgenomes derived from lineage-specific whole-genome triplication (WGT) exhibit differential fractionation^11,21^. The least fractionated subgenome is more likely to undergo gene conversion events^11^, and genes located in this subgenome show higher expression levels compared with their syntenic counterparts located in the medium-fractionated and most fractionated subgenomes^11^. Genes retained in the overfractionated subgenome are more likely to be targeted by 24-nt smRNAs and have higher transposon element (TE) coverage in their upstream regions, resulting in reduction of gene expression and driving subgenome expression dominance^21,22^.

However, biased fractionation between subgenomes is not always associated with the diploidization process following paleopolyploidization. The phenomenon of unbiased fractionation has been observed in some plants. *Camelina sativa*, a newly formed paleopolyploid (~5.5 Mya), has three undifferentiated subgenomes with similar gene number^23^. In poplar, two paleo-subgenomes derived from lineage-specific WGD show high similarity, suggesting that poplar may have originated from an autotetraploid ancestor^14^. Large-scale gene loss and divergence are not evident in the young allopolyploid *Brassica napus* formed ~7500 years ago; however, abundant homeologous exchanges have occurred since polyploidization^24^. In parallel, gene loss and expression bias between homoeologous gene pairs are rare in the evolution of the allotetraploid cotton (*Gossypium hirsutum*) genome formed ~1–1.5 Mya^25^. In addition, a recent study also revealed slow gene loss but rapid expression differentiation after WGD (~8 Mya) in common carp^26^.

Pear (*Pyrus bretschneideri* Rehd.) experienced a recent WGD (~30 Mya) event following an ancient γ hexaploidy event shared by core eudicots. However, it is not clear whether this recent polyploidy event in pear is an autopolyploidization or an allopolyploidization. Information concerning the evolutionary patterns of the two paleo-subgenomes descended from the recent WGD in pear is limited. Therefore, this study aimed to identify the two paleo-subgenomes of the pear genome and explore their evolutionary trajectory. Transcriptome and methylome data were used to investigate the evolutionary patterns of the two subgenomes. Our results show that the two subgenomes remaining in pear have evolved in an unbiased manner, suggesting that pear evolved from an autotetraploid ancestor.

## Materials and methods

### Data collection

Genome sequences and annotation files for Chinese white pear (*Pyrus bretschneideri* Rehd.) were obtained from the Pear Genome Project^27^. Genome data sets for peach (*Prunus persica*, v2.1) and woodland strawberry (*Fragaria vesca*, v1.1) were downloaded from Phytozome v12^28^.

### Identification of two paleo-subgenomes in pear

All-versus-all BLASTP was performed to search for paralogous gene pairs using whole-genome protein sequences of pear (E_value < 1e^−05^, m8 format). All-versus-all BLASTP was also used to search for orthologous gene pairs between pear and peach or strawberry. The MCScanX toolkit^29^ was used to identify intraspecies and interspecies syntenic blocks using BLASTP results and chromosomal locations of genes (match_score: 50, match_size: 5, gap_penalty: -1, overlap_window: 5, E_value: 1e^−05^, max gaps: 25)^30^.

False-positive interspecies syntenic blocks were removed from the MCScanX output according to two criteria: 1) blocks with E-value > 1e−10; 2) blocks with fewer than 10 gene pairs and more than 50% of gene pairs having E-values > 1e−10.

Pear experienced an ancient WGD (~140 Mya), shared with peach, and a lineage-specific WGD (~30 Mya) after splitting from peach. All interspecies syntenic blocks between pear and peach were identified, and the Ks value for each syntenic block was calculated. Ks peaks corresponding to the ancient WGD event and speciation event were inferred by fitting Gaussian mixture models to Ks value distributions of pear-peach syntenic blocks. Interspecies syntenic blocks with Ks values located in the Ks range corresponding to the ancient WGD event were removed.

Furthermore, tandem duplicate genes in the genomes of pear, peach and strawberry were identified using *duplicate gene classifier*, the core program of MCScanX. Paralogous or orthologous gene pairs that had experienced tandem duplication were excluded.

Syntenic blocks identified in the pear genome were grouped into two subgenomes (pear 1 and pear 2) according to a previously described method^12^. Homologous (best-match) pear chromosomal regions were assigned to subgenome 1 or subgenome 2 according to the number of singleton genes in each region. The chromosomal region with more singleton genes was assigned to subgenome 1, while the region with fewer singleton genes was assigned to subgenome 2. The detailed method for constructing the two subgenomes is described in the following example. Two regions of pear chr13 and chr16 were found to be collinear with the first half of peach chr1, while another two regions of pear chr8 and chr15 were found to be collinear with the second half of peach chr1. The two collinear pear chromosome regions were assigned to different subgenomes according to the number of singletons. The chromosomal region with more singletons was assigned to subgenome 1 (e.g., chr13 and chr15), and the other region was assigned to subgenome 2 (e.g., chr8 and chr16).

When a gene in the subgenomes of pear had a syntenic counterpart in peach and strawberry, respectively, this gene was defined as a high-confidence gene^12,15^. The high-confidence genes were used to investigate evolutionary patterns of gene expression level and DNA methylation between the two subgenomes.

### RNA-Seq library construction

Total RNA from leaf, fruit, petal, sepal, ovary, stem and bud was extracted using an RNAprep Pure Plant Kit (Polysaccharides & Polyphenolics-rich) (Tiangen, Beijing, China) following the manufacturer’s instructions and dissolved in RNase-free DNase I (Thermo, USA) to remove residual DNA. Total RNA was treated using oligo(dT) magnetic beads to purify mRNA, which was then fragmented using sonication. First-strand and second-strand cDNA was synthesized using random hexamer primers, and double-stranded cDNA was ligated to an A-tail and special sequencing adaptor (Illumina gene expression sample preparation kit, San Diego, CA). PCR was performed with Phusion High-Fidelity DNA polymerase, universal PCR primers and Index (X) Primer. The library preparations were sequenced on an Illumina HiSeq platform to generate 125 bp/150 bp paired-end reads (Novogene, Beijing, China). Leaves and fruits were replicated two times, and the other tissues were replicated three times.

### Bisulfite-seq library construction

Total genomic DNA was extracted from ovary with two biological replicates following a previously described protocol^31^. A total of 5.2 μg genomic DNA with 26 ng lambda DNA was fragmented to 200–300 bp by sonication, followed by end repair and adenylation. Lambda DNA was used to estimate the bisulfite conversion rate. The DNA fragments were then treated twice using an EZ DNA Methylation-GoldTM Kit (Zymo Research). Library concentration was quantified using a Qubit® 2.0 Fluorometer (Life Technologies, CA, USA) and quantitative PCR, and insert size was assayed using an Agilent Bioanalyzer 2100 system. The library preparations were sequenced on an Illumina HiSeq 2500 (Novogene, Beijing, China).

### RNA-Seq and Bisulfite-Seq data analysis

Trimmomatic (version 0.36) was used to remove adapter sequences and poly(A/T) tails, and filter low-quality reads (quality score < 15) from raw RNA-seq reads^32,33^. Kallisto was used to estimate the abundance levels of transcripts^34^. The Kallisto index was first built using whole-genome transcripts of pear, and then gene expression level (TPM, Transcripts Per Million) was estimated by applying the Kallisto quantification algorithm. Using the same workflow for intergenic sequences, an expression threshold (0.715) was calculated using the mean value of the median TPM values from seven different tissues. Information on RNA-seq samples used in this study is given in Supplementary Table S1.

Trim Galore! was used to remove low-quality reads from raw Bisulfite-seq reads (https://github.com/FelixKrueger/TrimGalore). The high-quality Bisulfite-seq reads were then mapped to the pear reference genome using Bismark v0.19.0 to estimate context-dependent (CpG, CHG, CHH) methylation level^35^. DeepTools was used to show the distribution of DNA methylation in different genomic regions^36^.

### Quantitative real-time PCR (qRT-PCR) analysis

To confirm expression differences between singleton and homeologous genes in the two subgenomes, a qRT-PCR experiment was performed. The procedure used to select gene sets of singletons and homeologs for qRT-PCR validation was as follows. In the ovary transcriptome, all singleton genes belonging to subgenome 1 (or subgenome 2) were sorted according to their expression level (TPM) in descending order. Then, the outliers beyond 1.5× interquartile range (IQR) in the boxplot were removed from TPM values set for singleton genes. Ten genes from top 10% of singleton genes were then randomly selected. Using a similar method, ten genes were chosen from the homeologs in subgenome 1 and ten from subgenome 2. In total, 40 genes were used for the qRT-PCR experiment, selected from four gene sets (Supplementary Table S2).

Total RNA was extracted from ovary and reverse-transcribed to cDNA as described above. Specific primers for 40 genes were designed using Primer Premier 5.0 software (PREMIER Biosoft International, USA), and the *Pyrus SNF* gene was selected as an internal reference. qRT-PCR was performed on a Lightcycle-480 (Roche). Relative expression levels were calculated using the 2^−ΔΔCt^ method^37^.

### Calculation of Ka and Ks values

A MCScanX downstream program was used to compute Ka and Ks values of orthologous syntenic gene pairs between pear (or peach) and strawberry using coding sequences and interspecies collinearity files as input files^29^. This program depends on ClustalW^38^ and Bio-perl (http://www.bioperl.org/).

### Statistical analysis

The Mann–Whitney *U* test was used to examine whether two sets of data (e.g., TPM values of subgenomes 1 and 2) differed significantly. *P*-values < 0.05 were considered significant.

## Results

### Syntenic relationships among pear, peach, and strawberry genomes

There are 42,341, 26,873, and 32,831 genes annotated in pear^27^, peach (*Prunus persica*)^39^ and woodland strawberry (*Fragaria vesca*)^40^, respectively. After removing false-positive syntenic blocks, 159 blocks from a total of 1290 syntenic blocks between pear and peach were discarded, and 140 blocks were removed from 1063 syntenic blocks between pear and strawberry. Using Ks values of pear-peach syntenic blocks, we calculated the Ks value of the speciation event (0.404) and ancient WGD event (1.37), respectively (Supplementary Fig. S1). Syntenic blocks derived from ancient WGDs were removed based on the ancient Ks peak. Genes involved in tandem duplication were also excluded.

After eliminating ancient syntenic blocks and tandem duplicate genes, we identified 824 syntenic blocks between pear and peach, including 11,108 peach genes (41.34% of whole-genome genes) and 16,509 pear genes (38.99%). In addition, 734 syntenic blocks between pear and strawberry were identified, containing 9416 strawberry genes (28.68%) and 14,132 pear genes (33.38%). A further 446 syntenic blocks were found between peach and strawberry, involving 12,627 peach genes (46.99%) and 14,915 strawberry genes (45.43%). The largest syntenic block was found between pear chr15 and peach chr1 and contained 427 collinear gene pairs, while the smallest syntenic block contained only five collinear gene pairs. The genome of pear showed greater collinearity with that of peach (61%) than that of strawberry (45%), corresponding to the closer phylogenetic relationship between pear and peach. Peach (or strawberry) has not experienced a lineage-specific WGD after its split from pear. Therefore, it was suitable to use peach as an outgroup species for identifying the two subgenomes derived from the recent WGD in pear. When using peach genes as a reference, a 2:1 syntenic relationship between pear and peach was found for 6203 (55% of all genes) peach genes, which was higher than the number of genes corresponding to other types of relationship (1% for 0:1, 36% for 1:1, 5% for 3:1, and <1% for 4:1). When using pear genes as a reference, a 1:1 syntenic relationship between pear and peach was dominant and was found for 14,859 (94% of all genes) pear genes. The above results suggest that the pear genome experienced a recent WGD after splitting from peach, resulting in a 2:1 syntenic pattern (Supplementary Fig. S2). The syntenic relationship between pear and peach was visualized using MCscan (Python version) incorporated in jcvi (https://github.com/tanghaibao/jcvi/wiki/MCscan-(Python-version))^41^.

### Unbiased fractionation and evolutionary patterns between the two subgenomes of pear

**Fig. 1:**
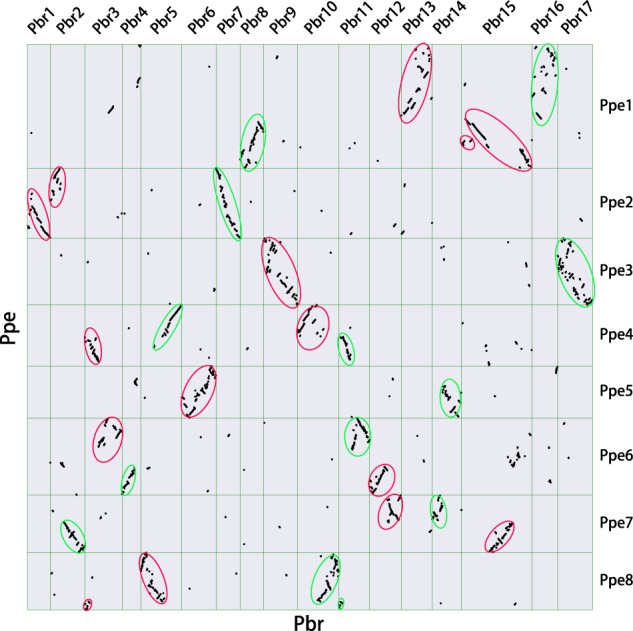
Whole-genome comparison between pear and peach. The *X* and *Y* axes represent the chromosomes of pear and peach, respectively. Each dot represents a collinear gene pair. The regions consisting of pear subgenome 1 in the dot plot are circled in purple, and the regions consisting of pear subgenome 2 are circled in green. Pbr: pear; Ppe: peach

Pear experienced a recent WGD, which occurred after the split of pear and peach^27^. Therefore, the peach genome can be used as an outgroup to identify the remnants of the two ancestral subgenomes derived from the recent WGD in pear^39,40^. Based on whole-genome comparison between pear and peach (Fig. 1), we reconstructed two paleo-subgenomes in pear. Reconstructed subgenome 1 (denoted as pear 1) included 2371 singleton genes, and subgenome 2 (denoted as pear 2) included 1752 singleton genes; 3126 homeologous gene pairs between subgenome 1 and subgenome 2 were identified (Supplementary Table S3, Supplementary Table S4).

**Fig. 2:**
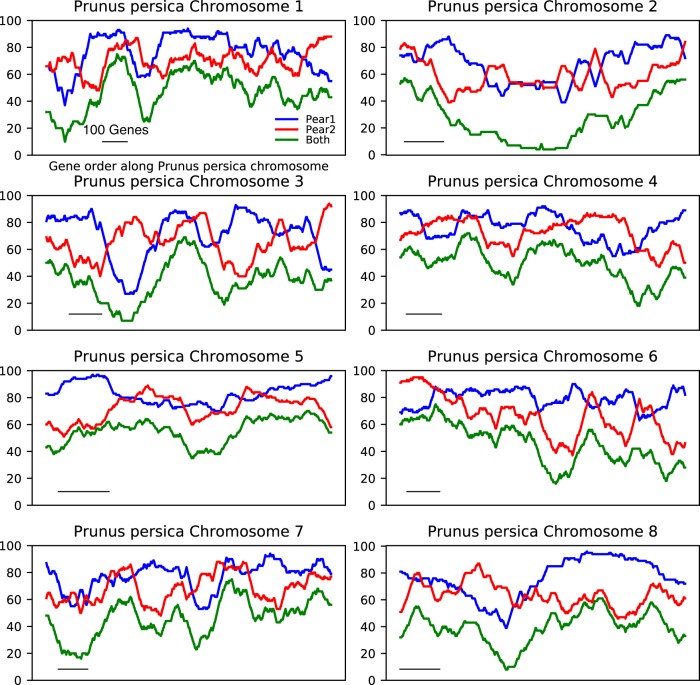
Fractionation pattern on each reconstructed pear paleo-subgenome corresponding to 8 peach chromosomes. The *X* axis indicates gene locations along each peach chromosome, and the *Y* axis indicates the proportion of orthologous syntenic genes retained in pear subgenome 1 (blue), subgenome 2 (red) and both subgenomes (green), corresponding to peach chromosomes. The percentage of retained orthologous genes in pear was calculated based on 100-gene sliding windows (black bars) along each peach chromosome. Tandem duplicate genes were excluded

We calculated the percentage of retained orthologous genes in pear based on a 100-gene sliding window along each peach chromosome. Neither subgenome 1 nor subgenome 2 was dominant for the number of retained pear genes (Fig. 2). Furthermore, we measured the rate of pear gene loss relative to that of all peach genes along each chromosome using the method described for poplar^14^. The results showed that pear subgenomes 1 and 2 had similar rates of gene loss, with no evident difference in gene loss rate between the two subgenomes (loss rate difference < 0.04) (Table 1). In addition, the minimum difference in gene loss rate between the two pear subgenomes was 0 along peach chromosome 1, and the maximum difference was only 0.04 along chromosome 5 or 8. The above results obtained from two different methods jointly support unbiased fractionation between the two pear subgenomes.

**Table 1:** Summary of the loss rate in pear’s two subgenomes

Peach		Pear			Loss rate difference
Peach chromosome	Peach Genes	Subgenome 1 loss rate	Subgenome 2 loss rate	Both loss rate	
Chr1	5883	0.85	0.85	0.79	0
Chr2	3303	0.83	0.85	0.75	0.02
Chr3	3161	0.79	0.80	0.70	0.01
Chr4	2897	0.76	0.78	0.69	0.02
Chr5	2464	0.79	0.83	0.69	0.04
Chr6	3662	0.80	0.83	0.74	0.03
Chr7	2720	0.71	0.73	0.62	0.03
Chr8	2721	0.78	0.82	0.71	0.04

Furthermore, we identified high-confidence genes from subgenome 1 and subgenome 2. If a gene in a pear subgenomic region had a syntenic counterpart in strawberry, we considered this gene a high-confidence gene. In total, we found 1396 and 975 high-confidence singleton genes in pear subgenome 1 and subgenome 2, respectively, and 1709 high-confidence homeologous gene pairs. The high-confidence singleton genes or homeologous gene pairs were used in the following analysis to guarantee the accuracy of results.

**Fig. 3:**
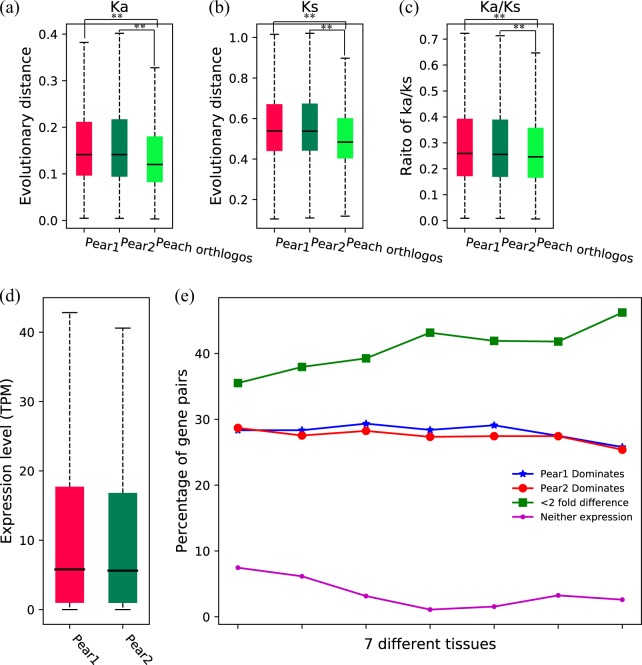
Comparison of evolutionary rates and expression patterns between pear subgenome 1 and subgenome 2. **a**–**c** Comparison of Ka, Ks, and Ka/Ks between genes in each pear subgenome and their orthologous genes in peach. Ka, Ks, and Ka/Ks values were estimated by pairwise comparison of orthologous genes between pear or peach and strawberry. **d**–**e** Comparison of overall expression level between the two subgenomes and the expression pattern of homeologous gene pairs in different pear tissues. Mann–Whitney *U* test was used for statistical analysis. *p-value < 0.05. **p-value < 0.01

We also calculated Ka, Ks, and Ka/Ks values between each high-confidence gene in pear’s two subgenomes and its syntenic counterpart in strawberry. The Ka, Ks, and Ka/Ks values of orthologous syntenic gene pairs between peach and strawberry were also computed. The genes in pear 1 and pear 2 evolved at similar evolutionary rates (*p*-value > 0.05, Fig. 3a–c). The genes in pear 1 and pear 2 had higher Ka, Ks, and Ka/Ks values than their syntenic counterparts in peach (*p*-value < 0.01), suggesting that genes in pear subgenomes have experienced more extensive mutation (Fig. 3a). The genes in pear 1 and pear 2 had small Ka/Ks ratios (<1), implying that they have experienced purifying selection (Fig. 3c).

Furthermore, we investigated the expression bias between the two subgenomes using high-confidence gene pairs and RNA-seq expression profiles from seven different tissues (Supplementary Table S1). The overall expression levels of homeologous genes between pear 1 and pear 2 showed no significant differences (*p*-value > 0.05) (Fig. 3d). If two members of a homeologous gene pair had more than a twofold difference in expression, we defined the member with higher expression as the dominant gene. We found that the percentage of homeologous genes with dominant expression in pear 1 was close to the percentage of homeologs with dominant expression in pear 2 in different tissues (Fig. 3e). A large number of homeologous gene pairs showed conserved expression levels, with a less than twofold expression difference. A small proportion of homeologous gene pairs in which both members had no expression was found in different tissues.

In addition, we detected DNA methylation levels of homeologous genes belonging to pear 1 and pear 2 in the gene body, the 3-kb region upstream of the transcription start site (TSS) and the 3-kb region downstream of the transcription termination site (TTS). The quality of methylation sequencing was estimated, with the bisulfite conversion rate in two replicates being higher than 99.8%. The Q30 and GC values met our analytical requirements (Supplementary Table S5).

**Fig. 4:**
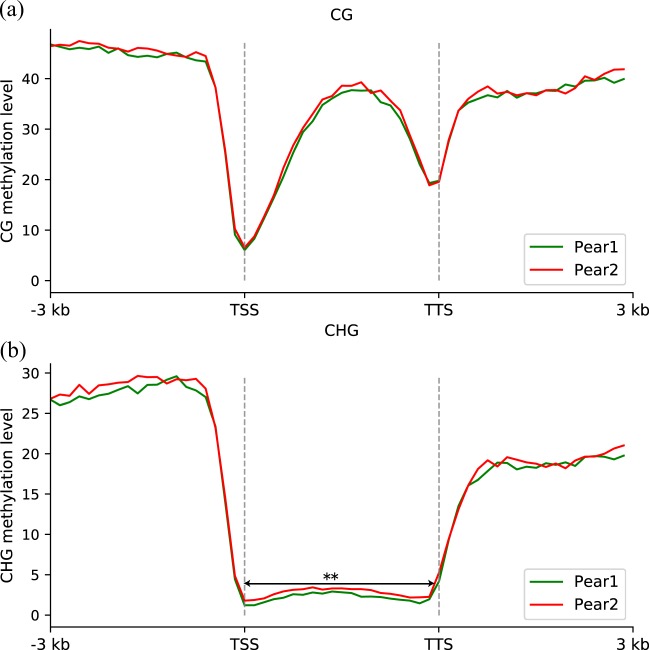
Comparison of DNA methylation levels of homeologous genes between pear subgenome 1 and subgenome 2. **a** The CG methylation levels of homeologous genes in the two subgenomes. **b** The CHG methylation levels of homeologous genes in the two subgenomes. TSS transcription start site, TTS transcription termination site. *p-value < 0.05. **p-value < 0.01, Mann–Whitney *U* test

We focused on CG and CHG methylation because these types are more prevalent than CHH methylation. Whole-genome bisulfite sequencing data from ovary was used (BioProject: PRJNA503323). To accommodate more potential methylation sites, we integrated the methylation sites identified from two biological replicates. The CG methylation levels between pear 1 and pear 2 showed no significant differences in the gene body, 5’ upstream region or 3’ downstream region (Fig. 4a). The CHG methylation level between pear 1 and pear 2 showed no significant bias in the 3’ downstream region or 5’ upstream region but showed significant differences in the gene body (*p*-value < 0.01), with pear 2 being overmethylated (Fig. 4b).

To confirm these results, we identified the methylation sites for each biological replicate using the same procedure. The results from the two biological replicates were identical for both CG methylation (Supplementary Fig. S3a, 3c, 3e) and CHG methylation (Supplementary Fig. S3b, 3d, 3f) and supported the results obtained by integrated analysis of the two biological replicates. These results provide evidence of unbiased DNA methylation patterns between the two subgenomes in pear.

### Divergent evolutionary patterns between singletons and homeologs within each subgenome

**Fig. 5:**
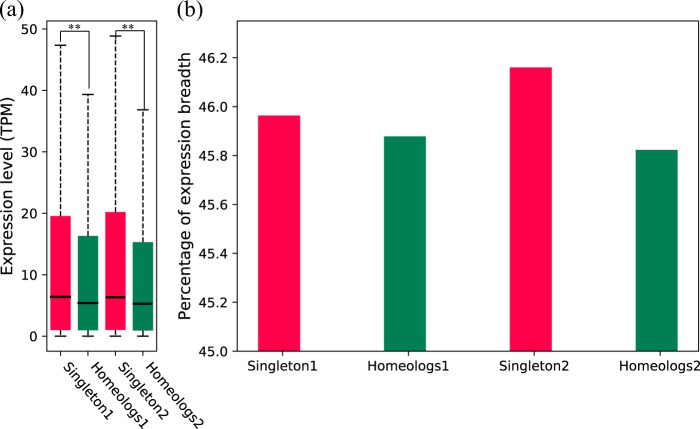
Comparison of expression level and expression breadth between singletons and homeologs. **a** Comparison of overall expression level between singletons and homeologs in each subgenome. **b** Comparison of percentage of expression breadth between singletons and homeologs. Mann–Whitney *U* test, *p-value < 0.05. **p-value < 0.01

The genes in pear 1 (or pear 2) without homeologous counterparts in pear 2 (or pear 1) were defined as singleton genes. Therefore, we distinguished two sets of genes in each pear subgenome: singleton genes and homeologous genes. The overall expression level of singletons was significantly higher than that of homeologs in both pear 1 and pear 2 (*p*-value < 0.01) (Fig. 5a). Moreover, we estimated average expression breadth for singletons and homeologs. The expression breadth for each gene was measured as the percentage of the seven tissues investigated in which this gene was expressed above the threshold level (0.715) (Fig. 5b). The results showed that singletons in both pear 1 and pear 2 have greater expression breadth than homeologs.

**Fig. 6:**
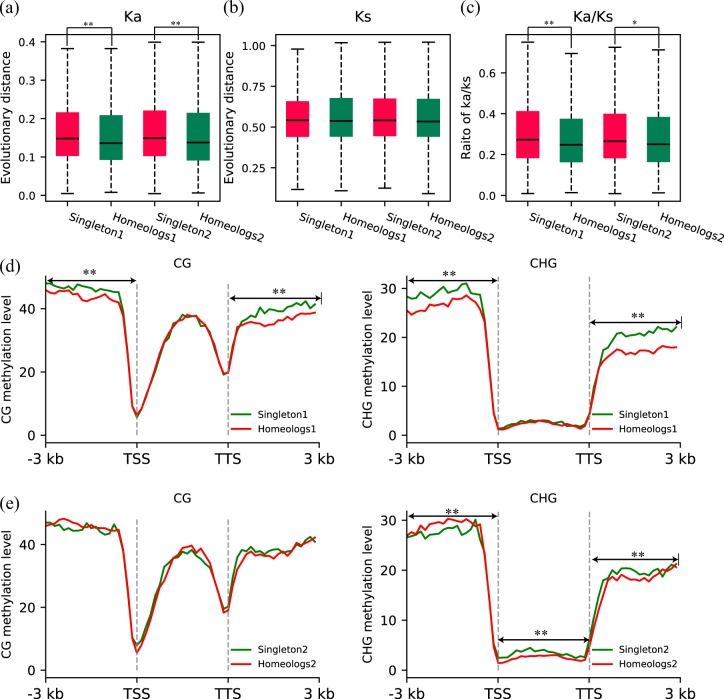
Comparison of evolutionary patterns and DNA methylation between singletons and homeologs. **a**–**c** Comparison of Ka, Ks, and Ka/Ks between singleton genes and homeologs. Ka, Ks, and Ka/Ks values were estimated by pairwise comparison of orthologous genes between pear or peach and strawberry. **d** Comparison of CG and CHG methylation levels between singletons and homeologs in pear subgenome 1. **e** Comparison of CG and CHG methylation levels between singletons and homeologs in pear subgenome 2. The regions with significant DNA methylation differences are indicated by a horizontal line with a two-way arrow. Singletons and homeologs are shown in green and red in **d** and **e**. Mann–Whitney *U* test: *p-value < 0.05. **p-value < 0.01

We further compared evolutionary rate and DNA methylation level between singletons and homeologous genes in each pear subgenome (Fig. 6). We compared Ka, Ks, and Ka/Ks values between these two sets of genes (Fig. 6a–c). In pear subgenomes 1 and 2, singletons had higher Ka values than homeologs (*p*-value < 0.01), indicating that singletons experienced more nonsynonymous mutations in their coding sequences (Fig. 6a). We found a similar distribution of Ks between singletons and homeologs, suggesting a similar evolutionary age for these two sets of genes (Fig. 6b). In pear subgenomes 1 and 2, singletons showed higher Ka/Ks ratios than homeologs (*p*-value < 0.01, *p*-value < 0.05), implying that singletons in both subgenomes evolved under stronger selective pressure than homeologs (Fig. 6c).

The CG and CHG methylation levels between singletons and homeologs in pear 1 showed significant differences in the 5’ upstream region and 3’ downstream region (*p*-value < 0.01), while no differences were found in the gene body region (Fig. 6d). In pear 2, we found significant CHG methylation differences in the gene body, 5’ upstream region and 3’ downstream region (*p*-value < 0.01) (Fig. 6e); however, CG methylation differences were not identified. To confirm the above results, we identified methylation sites for each biological replicate using the same procedure. The results from the two biological replicates were identical for CG methylation and CHG methylation (Supplementary Fig. S4) and supported the results obtained by integrated analysis of the two biological replicates.

**Fig. 7:**
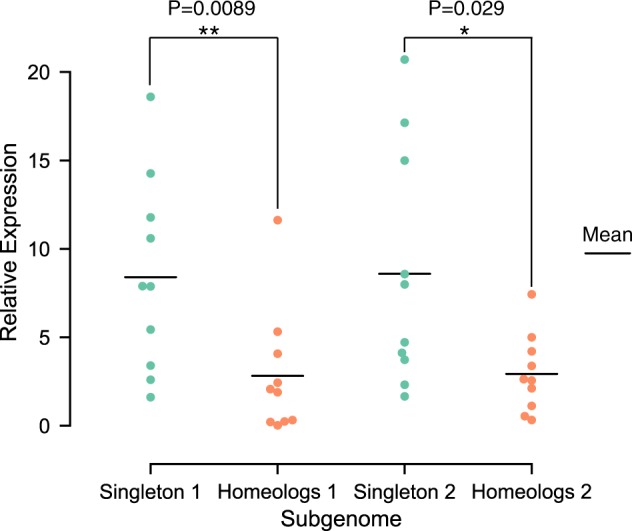
Comparison of relative expression level between singleton and homeologs by quantitative RT-PCR. The Y axis represents relative expression level. The mean relative expression in singleton and homeologs is marked using black bars. Mann–Whitney *U* test: *p-value < 0.05. **p-value < 0.01

To confirm expression differences between singletons and homeologs in each subgenome, a qRT-PCR experiment was performed. We randomly selected several singleton and homeologous genes (see details in Materials and methods) and compared the relative expression level between singletons and homeologs in each subgenome (Fig. 7). There was a significant difference (pear 1: *P* < 0.01; pear 2: *P* < 0.05) in relative expression level between singletons and homeologs, with observations based on transcriptome analysis. Mean values for singletons 1 and homeologs 1 were 8.4 and 2.82, respectively. Mean values for singletons 2 and homeologs 2 were 8.59 and 2.93, respectively. The results obtained from qRT-PCR and transcriptome analysis jointly supported singleton genes having higher expression levels than homeologs in each subgenome.

Although the evolution of the two subgenomes of pear has not been influenced by subgenome dominance, we found that the singletons and homeologs within each subgenome have evolved under a biased pattern.

## Discussion

Paleo-polyploidization is very common in the evolution of plant genomes^6,42–44^. Diploidization (or genome fractionation) often occurs rapidly following polyploidization events^8,9^. The two subgenomes derived from polyploidization may evolve in a biased or unbiased manner during the dipolyploidization process^13,45^. The phenomenon of subgenome dominance has been observed in some plant lineages, in which one of the two subgenomes exhibits a higher rate of gene loss and lower gene expression levels^12,46^. Paleo-allopolyploids are more likely to experience biased genome fractionation. Biased fractionation between two subgenomes was first found in the paleotetraploid *A. thaliana*^47^. A similar trend was also found in some other paleo-allopolyploids, such as maize^48^, sorghum^12^, brassica^10^ and cotton^18^. However, biased evolution between subgenomes is not the common rule following polyploidization, and the paleo-autopolyploids seem to have escaped from the constraints imposed by subgenome dominance. Genome fractionation between two subgenomes shows an unbiased pattern in paleo-autopolyploids and even some plant paleo-allopolyploid lineages, such as poplar^14^, soybean^15^, banana^45^, *Cucurbita maxima*, and *Cucurbita moschata*^16^.

In this study, unbiased fractionation between two subgenomes was found in the pear genome. This is consistent with observations in paleo-autopolyploids such as poplar^14^ and soybean^15^, which have two ancestral subgenomes with similar gene numbers. The evolutionary rate of homeologs between the two subgenomes in pear showed no significant difference, contrasting with results found in maize, in which the evolutionary rate of homeologs retained in the least fractionated subgenome was significantly slower than that of those in the overfractionated subgenome^15,20^. The genes retained in the overfractionated subgenome show lower expression levels and contribute less to phenotypic variation, eventually resulting in their loss owing to weak impact on fitness^12,19^. The stronger selective pressure imposed on genes in the overfractionated subgenome may cause more sequence mutations and decreased fitness resulting from reduction of gene expression, leading to a high rate of gene loss in the overfractionated subgenome^19,20^. However, expression dominance was not found between the two subgenomes in pear, which may be attributed to equivalent selection pressure imposed on the two pear subgenomes. Epigenetic modifications such as DNA methylation are closely related to gene expression^49,50^. In this study, we found no bias in CG methylation level between the two pear subgenomes, which may be associated with unbiased expression between them.

Duplicate genes tend to show tissue-specific expression, while singleton genes tend to show ubiquitous expression, with similar expression patterns to housekeeping genes^51,52^. The decreased expression breadth of duplicate genes can be explained by the subfunctionalization model, under which complementary loss of *cis*-regulatory elements between parent and daughter genes take place after gene duplication, facilitating the preservation of both partially expressed copies to maintain the full expression profile of the ancestral gene in different tissues and conditions^53,54^. In this study, divergent evolutionary patterns between singleton genes and homeologous genes within each pear subgenome were revealed. Singleton genes had higher expression breadth than homeologs in each subgenome, consistent with the subfunctionalization model. In addition, the greater expression breadth of singletons may be attributed to neofunctionalization. In each pear subgenome, the singletons evolved under stronger selection pressure than the homeologs and are therefore more likely to experience neofunctionalization, with one gene acquiring a new *cis*-element, leading to expression in a new tissue or physiological condition^55–57^. In addition, we found that the mean expression level of singleton genes was significantly higher than that of homeologs. This is in consistent with the observation that reduction in expression of duplicated gene copies occurs frequently after gene duplication^58^. The reduced expression of homeologous genes can be partially explained by the dosage subfunctionalization model, under which two gene copies are preserved over a long time by partitioning of the total expression dosage level of the progenitor gene^59^. Moreover, differential methylation levels between singletons and homeologs in pear subgenomes were identified, which may be associated with expression differences between them. Higher DNA methylation in the 5’ upstream region of the TSS and the 3’ downstream region of the transcription termination site will result in lower gene expression^50,60^. However, lower expression of singletons was not found in this study, although a higher CG or CHG methylation level of singletons was found in the 5’ upstream or 3’ downstream regions.

In summary, we found no significant bias in gene loss rate, evolutionary rate, expression level or DNA methylation level between the two subgenomes of pear. The results of this study suggest that pear may have originated from an autotetraploid ancestor. The unbiased evolution between the two subgenomes has persisted over 30 million years after the recent WGD in pear. This study enhances our understanding of the postpolyploidization diploidization process in pear and other plants.

## Supplementary information

Supplementary Table S1

Supplementary Table S2

Supplementary Table S3

Supplementary Table S4

Supplementary Table S5

Supplememtary Fig. S1-S4

## Data Availability

The transcriptome raw reads from seven different pear tissue and methylome raw reads from ovary have been deposited at NCBI (https://www.ncbi.nlm.nih.gov/bioproject/) under accession numbers PRJNA498777 and PRJNA503323.
