# Supplementary material for: Unbiased subgenome evolution following a recent whole-genome duplication in pear (Pyrus bretschneideri Rehd.)
Source: Hortic Res. 2019 Mar 1;6:34. doi: 10.1038/s41438-018-0110-6 (PMC6395616; doi:10.1038/s41438-018-0110-6)
Supplement: Supplementary file 2 — Supplementary Table S2 [file 41438_2018_110_MOESM2_ESM.docx]

**Supplementary Table S2** qRT-PCR specific primers used in this study.

|  | Gene ID | Forward primer | Reverse primer |
| --- | --- | --- | --- |
| Reference | SNF | GATGGTGCTATGAAGATGCCAAATGT | TCCCGAGCATCACGATAGATTCAC |
| Singleton 1 | Pbr026235.1 | TCATCCAGGTTTTCGGGTCCA | TCTCGGGTTGTAACTCGGGTCG |
|  | Pbr004378.1 | CTCTTCTCCCTCACCTCCGTTCC | AGCAATTCGTCGTTCTGAGCCG |
|  | Pbr007689.1 | ACAGGCGATGGGAAAGTGGCT | GGAAGGCGGAGGTACGCAGAGT |
|  | Pbr008780.1 | GCGAAAGTCGGCGTGGAGG | AAGAACGTGGCGGAGCGGTA |
|  | Pbr042857.1 | CCCAATAGTCCCGCCACCG | ATCCGAACCCCGATCCCTCA |
|  | Pbr011483.1 | GGTATAGCTCGCCAGCGTCAAG | GGGGAATAAACACGGCTGAGGAT |
|  | Pbr011483.1 | TGCTGGGGTTCTCCGTCAATGT | GGGGAATAAACACGGCTGAGGAT |
|  | Pbr019854.1 | ATGAG CAAAGATGACGACGACCC | TCCGTCCACCGCCGAGAAT |
|  | Pbr004310.1 | CTTTGTGCTGCTGCTTTGGTCG | ATGTTGGTCTCCACGGAAGCGA |
|  | Pbr018801.2 | CTGTGCTTTGTGATGCCGAGGTT | GCTTGAGAGAGAGGGGGGATGAA |
| Homeologs 1 | Pbr019284.1 | GGAAACAGATAGCGATGGCAGGTC | GGTAAACGGTGTCCTCCGCATC |
|  | Pbr003187.1 | GCCTTGGCTTAGGAATCCTTTGC | ATCCCCTGTCCTCAAACCCTGTG |
|  | Pbr011437.1 | TACTGACGGGGAGGCTGAGGC | ATCTCCACCGACGACCCAAAACT |
|  | Pbr004946.1 | GAAAGAAGGCGAGGGTCTGGGA | GAACGGCGACGACACAGAAACAT |
|  | Pbr037017.1 | TCAAGGAAGTTGGAGTGGTGGCA | CTTCTCCTTCATCCTGTCAACGGC |
|  | Pbr000006.2 | TAAGGCACTGGCGATGACGATAAT | AACTGCTCCCCTTCCTCTGTGC |
|  | Pbr010517.1 | GGCGGCAACAGCAGCAAC | CGAAGAAGTCGGAATCGACGGA |
|  | Pbr022198.1 | TGAACGCTTGTCGGTGTCATCC | CACCAGGAACGCTGGGTCAGTAA |
|  | Pbr011143.1 | CGTCAACTTGGTTTGCTTCTCCCT | CCAACAACCCAGGTCGTTTCTCA |
|  | Pbr020344.1 | TACGCAACTTCTGGGGCTACGC | CCAATCTTCTGCCATCCACATCCT |
| Singleton 2 | Pbr041934.1 | TGTGAGTGAGACGCTGGCTTTTG | GCTGCGGAGGCTCGTAGATGA |
|  | Pbr023771.1 | GAGGAAGGAAAGCCAGATGCCC | GCTGCGTTGACTGCGATGGA |
|  | Pbr026566.1 | CCCGTCCAACCCCAGCAAC | CCGCACGCGATCTCCACAA |
|  | Pbr006550.1 | ATAAGGGCATTGTTGTGCTCGTCA | GTTAACTTTGGGTCCGCCAGGAT |
|  | Pbr026225.1 | AAGAAGAGGTGTTTGGACGCTTCA | AGACGCCGTGGACTGGGAGA |
|  | Pbr038227.1 | CGCCGCCCAATCTCGTTCT | ACACCCATCGAGGAGGATCGTC |
|  | Pbr038422.1 | ACAACACCGCCACCAAAATCG | GCCACAGCTTGACCTGAACCCT |
|  | Pbr026212.1 | CCGATGGCAGCACCTTGTCC | GGCGGTTTAGCCGGTTTGG |
|  | Pbr018476.1 | AGAGGCTTCAACAAGAACTGGCTG | ACCCCAAAATAATGCCAATCAAGC |
|  | Pbr041938.2 | AGGTGCTCTTAGGGGAGGTCGTG | TGGTCATTCTGTCCCTTTCCTGTG |
| Homeologs 2 | Pbr024972.1 | TCACTCCACCATACCCAAAAGGC | CTTCCGAAATGTCGCTTCCAATC |
|  | Pbr026533.1 | ACTCCAACCACCGCCGAACA | AGCTGCTTGCCGCAATTCGT |
|  | Pbr011601.1 | GATTCCAAAGGACTGCCCACAAA | ATTACCGTTGTTGTTTGACCGCAG |
|  | Pbr038899.1 | GGGGCAAGATTGATGCGGACA | CGAGTCTCCAGTCGCGAAATCTAT |
|  | Pbr041669.1 | GCAATTCTGCACCGGCGAGTA | CGCAAGTGATAAAGAACTGGCTCC |
|  | Pbr019449.1 | GGCAAGGCTGGGCTTACTCGA | CATTTGGCATTAGCAAGACGGTGA |
|  | Pbr007154.1 | TCGACGTGGTTACTCATGTTCCAA | GTTGTCACCAGGACACAGCAGGTT |
|  | Pbr013932.1 | GGCAGGGGCAGCCGATTAG | GAGACGGCATTGCTCCTTTCG |
|  | Pbr007339.1 | TGGAGCAGGGAAGATCAAGCAGT | CCCAACAGCATATCCTGCATCCT |
|  | Pbr024556.1 | TGAGGATCAGATGGTCCCAAGCAT | GGTGGCGGAAGAGCATTGTTATGA |
