# Supplementary material for: Unbiased subgenome evolution following a recent whole-genome duplication in pear (Pyrus bretschneideri Rehd.)
Source: Hortic Res. 2019 Mar 1;6:34. doi: 10.1038/s41438-018-0110-6 (PMC6395616; doi:10.1038/s41438-018-0110-6)
Supplement: Supplementary file 4 — Supplementary Table S4 [file 41438_2018_110_MOESM4_ESM.docx]

**Supplementary Table S4 The percentage of singleton genes and homeologous genes in each subgenome.**

| **Peach chromosome** | **Subgenome 1** | | | **Subgenome 2** | | | **Homeologous gene pairs** | |
| --- | --- | --- | --- | --- | --- | --- | --- | --- |
|  | **Chromosome ID** | **Singleton genes** | | **Chromosome ID** | **singleton genes** | |  |  |
|  |  | **Number** | **%** |  | **Number** | **%** | **Number** | **%** |
| Chr1 | Chr13,Chr15 | 350 | 28.88 | Chr16, Chr8 | 302 | 24.92 | 560 | 46.20 |
| Chr2 | Chr1, Chr2 | 313 | 38.54 | Chr7 | 261 | 32.14 | 238 | 29.31 |
| Chr3 | Chr9 | 311 | 32.63 | Chr17 | 287 | 30.11 | 355 | 37.25 |
| Chr4 | Chr3, Chr10 | 257 | 30.05 | Chr5, Chr11 | 196 | 22.14 | 432 | 48.81 |
| Chr5 | Chr6 | 206 | 33.55 | Chr14 | 85 | 13.84 | 323 | 52.61 |
| Chr6 | Chr3, Chr12 | 307 | 32.70 | Chr4, Chr11 | 199 | 21.12 | 435 | 46.17 |
| Chr7 | Chr12, Chr15 | 321 | 31.01 | Chr2, Chr14 | 236 | 22.80 | 478 | 46.18 |
| Chr8 | Chr3, Chr5 | 306 | 38.39 | Chr10, Chr11 | 186 | 20.74 | 305 | 38.27 |
