# Supplementary material for: Unbiased subgenome evolution following a recent whole-genome duplication in pear (Pyrus bretschneideri Rehd.)
Source: Hortic Res. 2019 Mar 1;6:34. doi: 10.1038/s41438-018-0110-6 (PMC6395616; doi:10.1038/s41438-018-0110-6)
Supplement: Supplementary file 5 — Supplementary Table S5 [file 41438_2018_110_MOESM5_ESM.docx]

| Sample | Raw reads | Clean reads | Total rates | Q30 (%) | GC (%) | BS conversion rate (%) | Mapped rates | Mapping rate(%) |
| --- | --- | --- | --- | --- | --- | --- | --- | --- |
| O1 | 69514996 | 68579805 | 68579805 | 93.98 | 22.62 | 99.898 | 33953861 | 49.51 |
| O2 | 69765952 | 68692558 | 68692558 | 93.58 | 22.42 | 99.875 | 33707438 | 49.07 |

**Supplementary Table S5 Data on Bisulfite-Seq reads for pear ovary sample with two replicates**

O1/2 represents pear ovary replicate 1/2
