# Supplementary material for: Unbiased subgenome evolution following a recent whole-genome duplication in pear (Pyrus bretschneideri Rehd.)
Source: Hortic Res. 2019 Mar 1;6:34. doi: 10.1038/s41438-018-0110-6 (PMC6395616; doi:10.1038/s41438-018-0110-6)
Supplement: Supplementary file 6 — Supplememtary Fig. S1-S4 [file 41438_2018_110_MOESM6_ESM.docx]

**Supplememtary Fig. S1-S4**


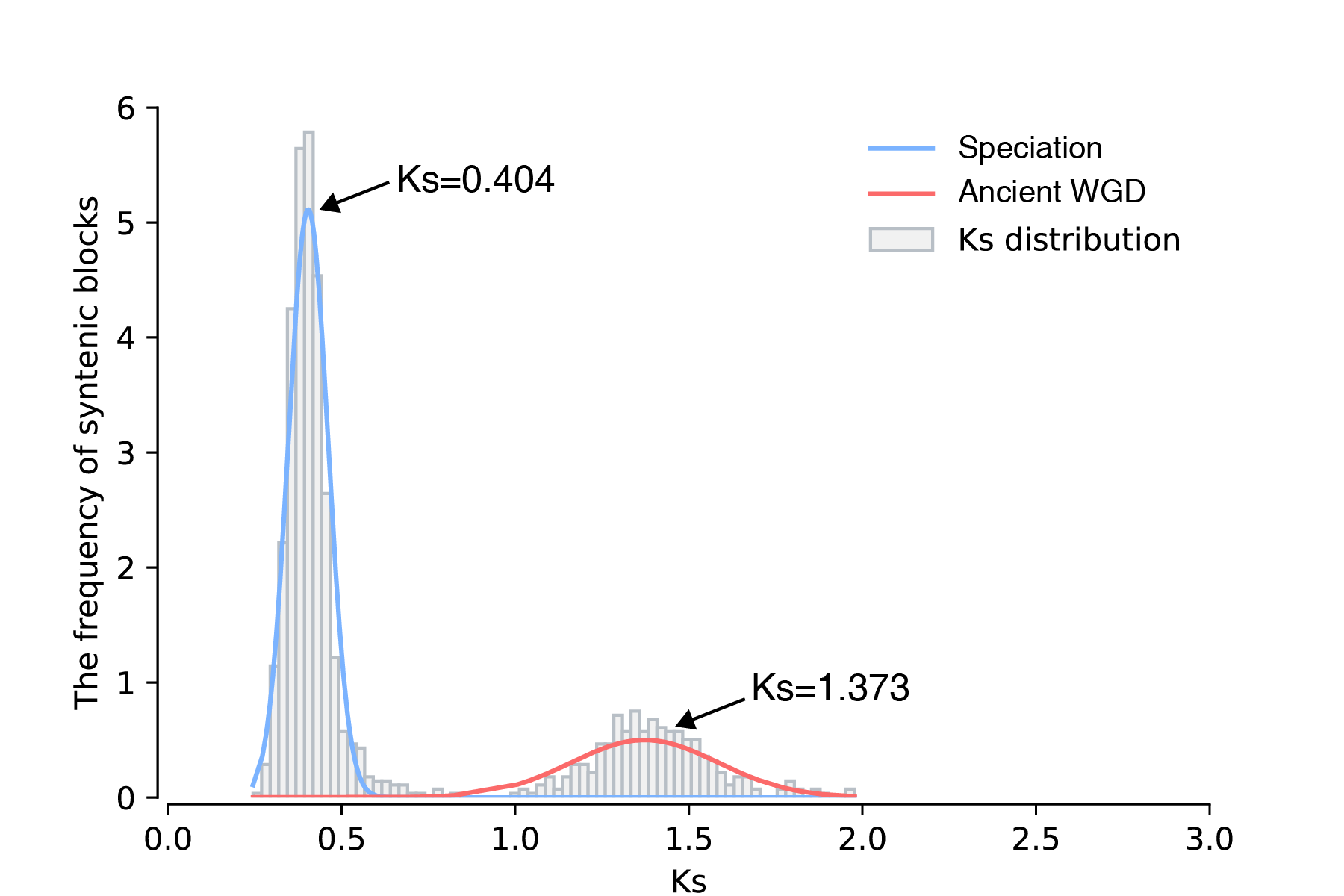


**Supplementary Fig. S1 Ks distribution of syntenic blocks between pear and peach.** The blue line indicates a speciation event, and the red line indicates an ancient WGD event.


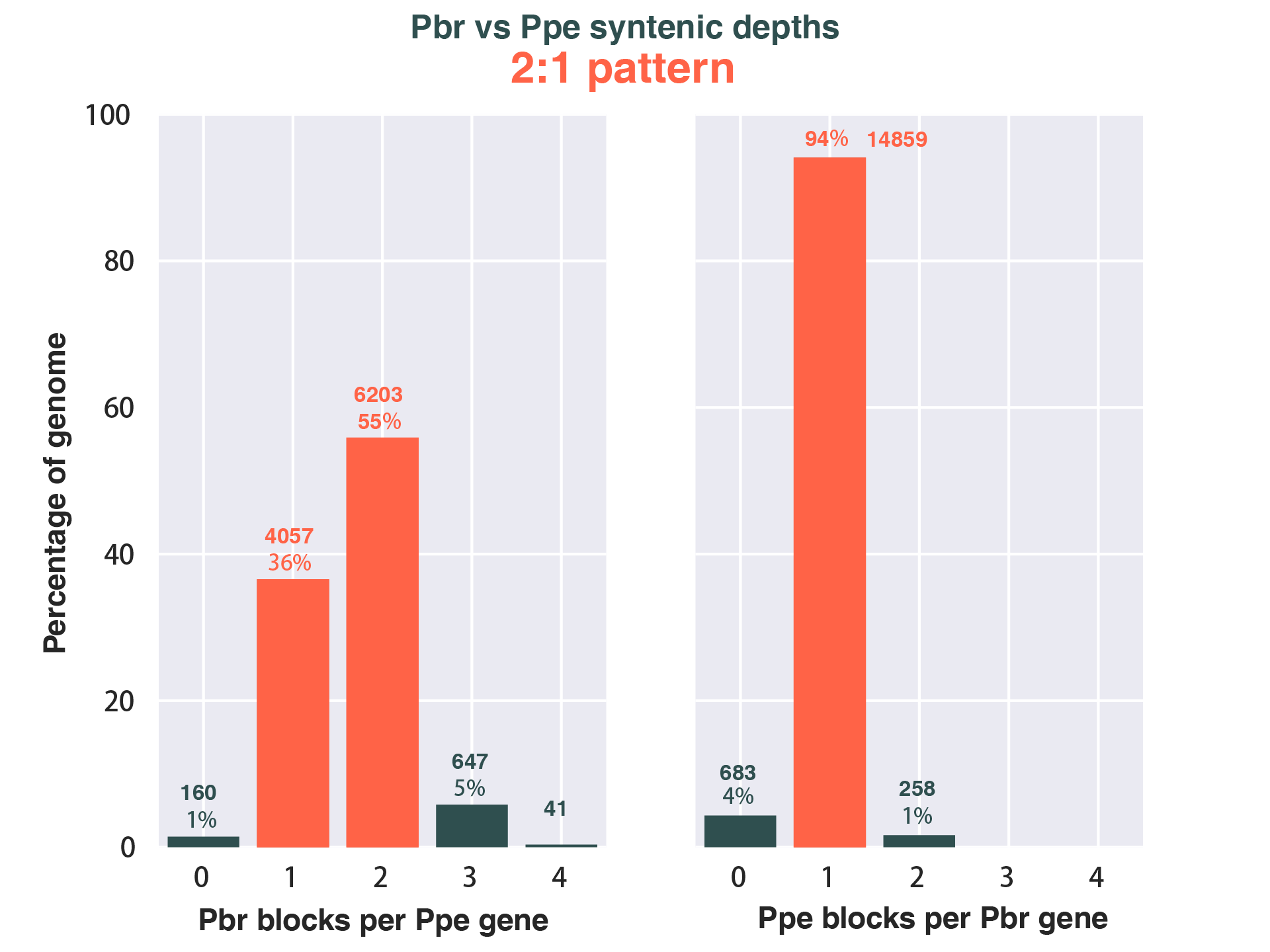


**Supplementary Fig. S2** **Syntenic relationship between pear and peach genome.** Pbr: pear; Ppe: peach.


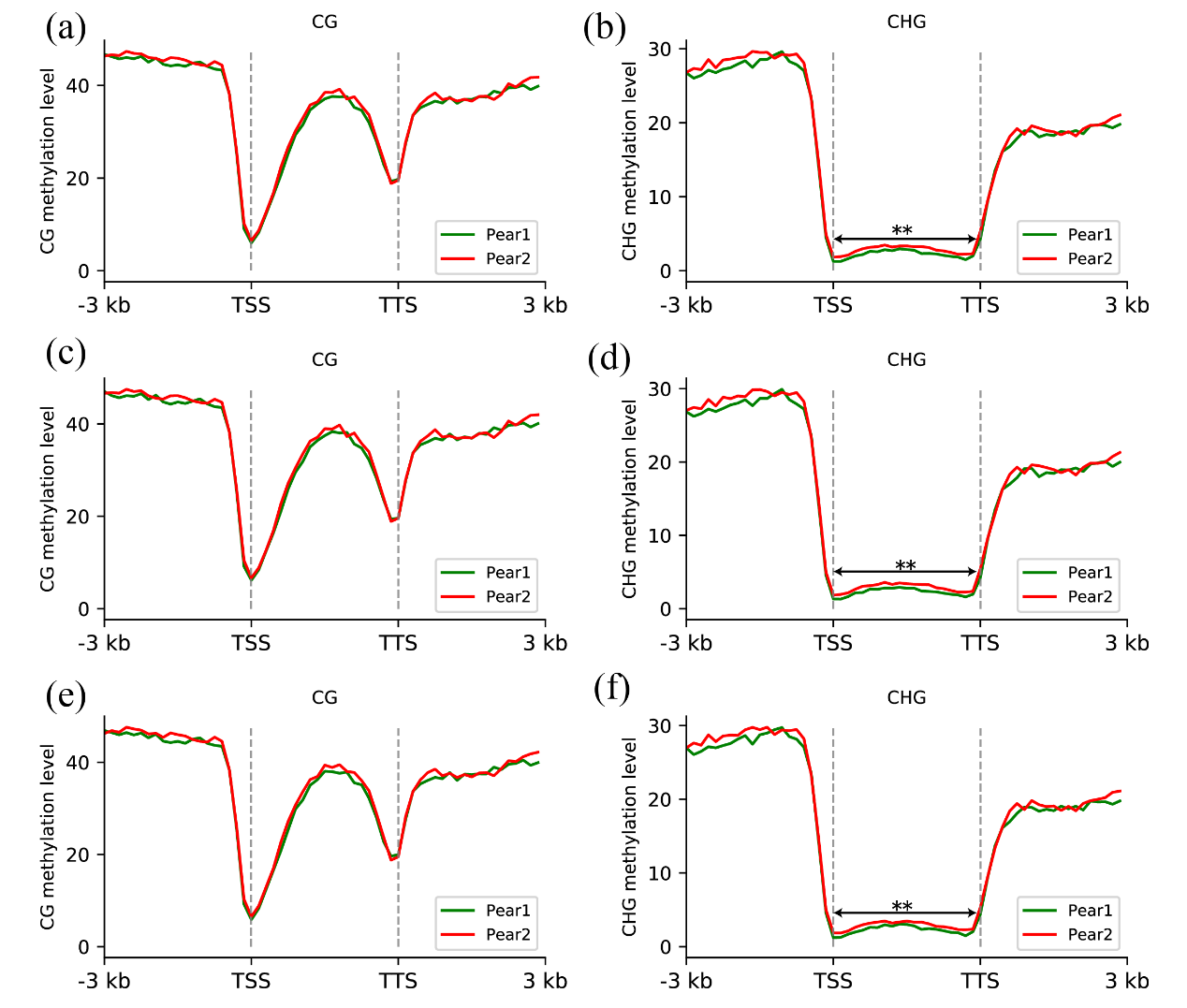


**Supplementary Fig. S3** **Comparison of DNA methylation levels between pear 1 and 2.** (**a-b**) CG and CHG methylation levels between pear 1 and 2 obtained from integrated analysis. (**c-d**) CG and CHG methylation levels between pear 1 and 2 obtained from biological replicate 1. (**e-f**) CG and CHG methylation levels between pear 1 and 2 obtained from biological replicate 2. Mann-Whitney U test: *, p-value <0.05. **, p-value<0.01.


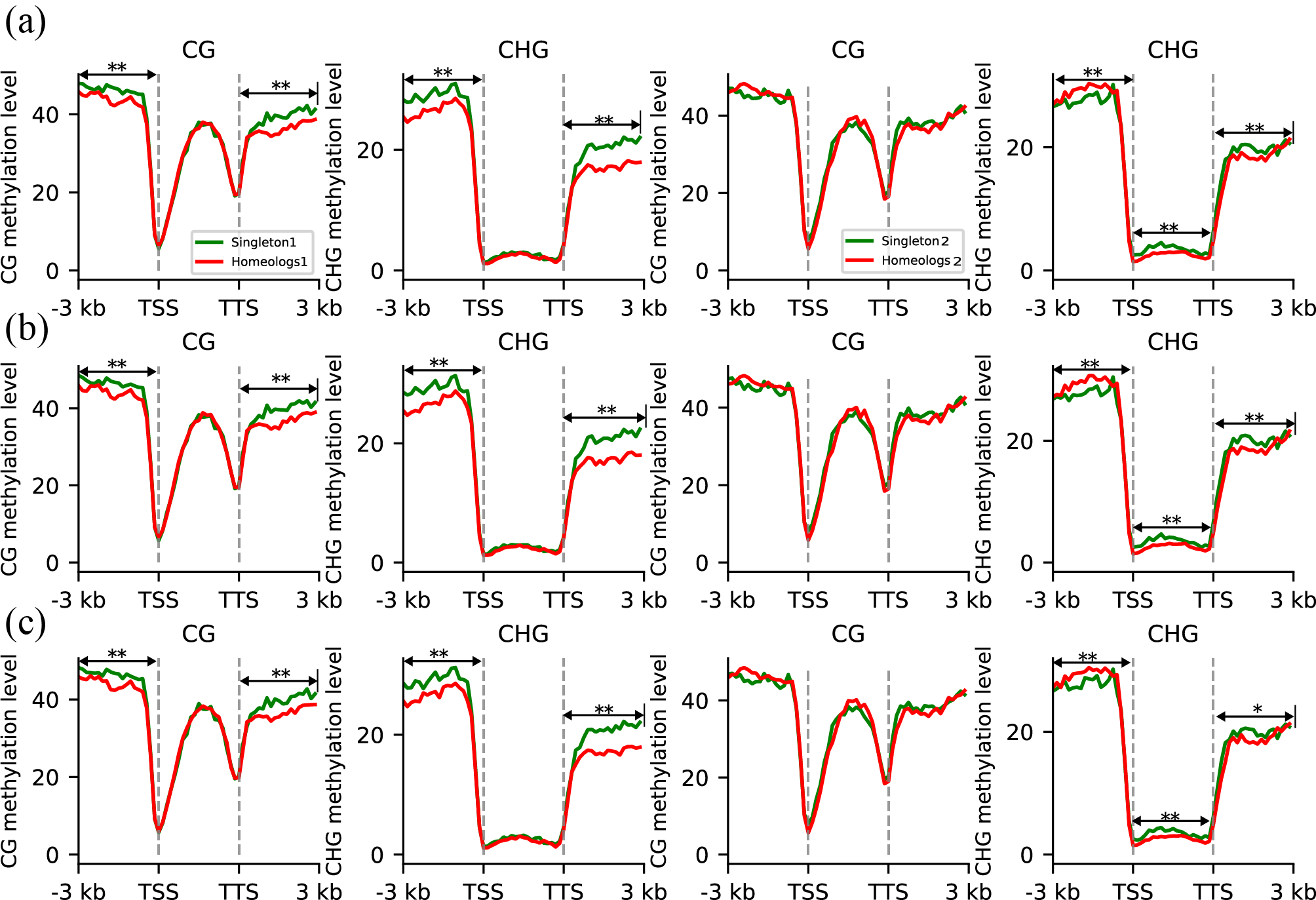


**Supplementary Fig. S4 Comparison of DNA methylation levels between singletons and homeologs.** (**a**) CG and CHG methylation levels between singleton and homeologs obtained from integrated analysis. (**b**) CG and CHG methylation levels between singleton and homeologs obtained from biological replicate 1. (**c**) CG and CHG methylation levels between singleton and homeologs obtained from biological replicate 2. Mann-Whitney U test: *, p-value <0.05. **, p-value<0.01.
